# The sGC stimulator BAY-747 and activator runcaciguat can enhance memory in vivo via differential hippocampal plasticity mechanisms

**DOI:** 10.1038/s41598-022-07391-1

**Published:** 2022-03-04

**Authors:** Ellis Nelissen, Nina Possemis, Nick P. Van Goethem, Melissa Schepers, Danielle A. J. Mulder-Jongen, Lisa Dietz, Wiebke Janssen, Michael Gerisch, Jörg Hüser, Peter Sandner, Tim Vanmierlo, Jos Prickaerts

**Affiliations:** 1 Department of Psychiatry and Neuropsychology, School for Mental Health and Neuroscience (MHeNS), Maastricht University, Universiteitssingel 50, 6229 ER Maastricht, The Netherlands; 2 Neuro-Immune Connect and Repair Lab, Biomedical Research Institute, Hasselt University, 3500 Hasselt, Belgium; 3 Bayer AG, Pharmaceuticals R&D, Pharma Research Center, 42113 Wuppertal, Germany; 4 Hannover Medical School, 30625 Hannover, Germany

**Keywords:** Learning and memory, Molecular neuroscience, Neuro-vascular interactions, Dementia

## Abstract

Soluble guanylate cyclase (sGC) requires a heme-group bound in order to produce cGMP, a second messenger involved in memory formation, while heme-free sGC is inactive. Two compound classes can increase sGC activity: sGC stimulators acting on heme-bound sGC, and sGC activators acting on heme-free sGC. In this rodent study, we investigated the potential of the novel brain-penetrant sGC stimulator BAY-747 and sGC activator runcaciguat to enhance long-term memory and attenuate short-term memory deficits induced by the NOS-inhibitor L-NAME. Furthermore, hippocampal plasticity mechanisms were investigated. In vivo, oral administration of BAY-747 and runcaciguat to male Wistar rats enhanced memory acquisition in the object location task (OLT), while only BAY-747 reversed L-NAME induced memory impairments in the OLT. Ex vivo, both BAY-747 and runcaciguat enhanced hippocampal GluA1-containing AMPA receptor (AMPAR) trafficking in a chemical LTP model for memory acquisition using acute mouse hippocampal slices. In vivo only runcaciguat acted on the glutamatergic AMPAR system in hippocampal memory acquisition processes, while for BAY-747 the effects on the neurotrophic system were more pronounced as measured in male mice using western blot. Altogether this study shows that sGC stimulators and activators have potential as cognition enhancers, while the underlying plasticity mechanisms may determine disease-specific effectiveness.

## Introduction

Evidence is accumulating that the second messenger molecule cyclic GMP (cGMP) is important in memory processes in general and long-term potentiation (LTP), the physiological correlate of memory, in particular^1^. One approach to increase cGMP is to enhance its production by targeting the enzyme soluble guanylate cyclase (sGC). sGC is a heterodimer comprised of an α-subunit and a β-subunit, and is localized in the cytoplasm. The β-subunit contains a so-called N-heme-nitric oxide binding domain (H-NOX), which has heme (Fe^2+^) binding properties^2^. Under physiological conditions, sGC activity is stimulated by nitric oxide (NO) derived from the nitric oxide synthases (NOS) endothelial (eNOS) and neuronal (nNOS). The binding of Fe^2+^ to His105 within this H-NOX domain is essential for the interaction between NO and sGC and the subsequent catalyzation of a cyclization reaction, converting guanosine triphosphate (GTP) into cGMP^3,4^. When oxidized, e.g. by reactive oxygen or nitrogen species, the ferrous Fe^2+^ group can be converted into a ferric Fe^3+^ group, which is thought to conformationally open up the binding pocket of the Fe^3+^ heme group within the β-subunit, and facilitate heme-loss of sGC^5,6^. Since the heme-group is needed for NO binding, and ferric heme does not bind NO well^5^, both the Fe^3+^-containing sGC (Fe(III)sGC) and the heme-free enzyme (apo-sGC) cannot be stimulated by NO and are thereby rendered inactive leading to disruption of NO–sGC–cGMP signalling.

Targeting sGC to enhance cGMP production can be done in two different ways: using an sGC stimulator that acts on the Fe^2+^-bound active sGC (Fe(II)sGC), or using an sGC activator that specifically targets oxidized Fe(III)sGC and apo-sGC^7^. Direct NO-independent sGC stimulation was first demonstrated in 1994, when Ko and colleagues reported a synthetic benzylindazole compound called YC-1, as a stimulator of cGMP synthesis^8^, which was subsequently verified to be a direct sGC stimulator^9,10^. YC-1 was characterized as a direct NO-independent, but heme-dependent, sGC stimulator^10^ and it was shown that treatment with YC-1 improves cognitive function in aged rats back to that of the level of adult rats^11^. These results provided a first indication that stimulation of sGC can be considered as a target to treat cognitive impairments in neurological and psychiatric disorders. Later studies showed that sGC stimulators riociguat and vericiguat could enhance long-term and short-term memory in rodents^12,13^. Yet the memory enhancing effects could most likely be attributed to effects on the cerebromicrovasculature, since both riociguat and vericiguat show very limited to no brain penetration and riociguat particularly attenuated the memory impairment effects of the cerebral vasoconstrictor sumatriptan^12^. Riociguat did not enhance memory in a clinical study^14^, yet the effective dose could have been missed in this study due to the narrow therapeutic window of riociguat. Recently, a study in mice was performed with CY6463, a novel sGC stimulator capable of crossing the blood–brain barrier, and the results indicated central effects of CY6463 (e.g. by enhancing the hypothalamic BDNF levels) and an improvement of memory performance, in contrast to vericiguat^15^. This study especially showed the promise of sGC as a target for cognition enhancement with brain-penetrant stimulators.

In addition to these promising effects of sGC stimulators, research has also shown that in a stroke model under acute oxidative stress, sGC activity is decreased in mouse homogenates of the cortex and basal ganglia, most likely due to oxidation of Fe(II)sGC and subsequent heme loss^16^. This suggests that under such particular conditions, sGC activators would be the go-to treatment for inducing neuroprotection. However, little is known about the differential and/or overlapping effects between sGC stimulators and activators on neuroprotection and synaptic plasticity, and the implications for neurodegenerative diseases with a deficient NO–sGC–cGMP signalling system such as Alzheimer’s disease and vascular dementia^7,17–19^. We recently showed ex vivo that the sGC stimulator vericiguat enhanced GluA1-containing AMPA receptor (AMPAR) mobilization to the membrane surface in mouse hippocampal slices, in a chemical LTP protocol mimicking memory acquisition processes^13^. Increased mobilization to the membrane and increased expression of the GluA1 subunit might be linked to increased synaptic plasticity and memory processes. In particular, transient AMPAR mobilization might be linked to acquisition, while elevated synthesis of AMPARs could explain longer lasting effects as improved consolidation^20^. Likewise, neurotrophic signalling in the hippocampus is important for synaptic plasticity underlying memory formation, and immediate measures such as BDNF splicing and more prolonged lasting effects such as BDNF gene expression could both be linked to improvements in memory^21–23^. Yet, in vivo treatment with sGC stimulator riociguat failed to enhance BDNF protein expression, nor did it affect AMPA receptor dynamics in the hippocampus of mice. However, this could also be related to the very limited ability of riociguat to cross the blood–brain barrier^12^, a property shared with vericiguat^13^. To our knowledge, no memory studies thus far have been performed with an sGC activator. In sum, the specific working mechanisms of sGC stimulators and activators and their effects within the central nervous system have thus far not been elucidated and studies with sGC activators on cognitive function are still elusive.

Therefore, the present study aims at identifying the effects of the brain-penetrant sGC stimulator BAY-747 and brain-penetrant sGC activator runcaciguat on memory performance and hippocampal plasticity in rodents in head-to-head studies. The object location task (OLT) was used to determine the effects of BAY-747 and runcaciguat on acquisition processes of long-term memory, and short-term memory impairments induced by the NOS inhibitor L-NAME. The effects of BAY-747 and runcaciguat on AMPAR dynamics as a measure for hippocampal plasticity was measured in an acquisition-like chemical LTP model ex vivo. Similarly, the effects of BAY-747 and runcaciguat were measured on AMPAR dynamics and the neurotrophic system during memory acquisition processes in vivo as a measure for hippocampal plasticity.

## Methods

### Animals

For the in vivo studies, all experimental procedures were approved by the local ethical committee of Maastricht University for animal experiments in accordance with governmental guidelines (approval codes: 2012-063 and 2018-006; Licensed animal ethical committee Ministry of VWS, GZBIVVB981845). For the long-term memory studies, two cohorts of twenty-four 3-month-old male Wistar rats (Charles River, Sulzfeld, Germany) were used (average body weight at the beginning of the study: cohort 1 BAY-747: 292 g; cohort 2 runcaciguat: 334 g). For the L-NAME studies, forty-eight 4-month-old male Wistar rats were used separated into two batches of twenty-four rats (average body weight at the beginning of the study: batch 1: 541 g; batch 2: 440 g). For the in vivo plasticity study, twenty-four 3-month-old male C57BL/6 mice (Charles River, Sulzfeld, Germany) were used (average body weight at the beginning of the study: 29.1 g). The animals were housed as described previously^13^. In brief, all animals were housed individually in a standard IVC cage system on sawdust bedding in an air-conditioned room (about 20 °C). They were kept under a reversed 12/12 h light/dark cycle (lights on from 19.00 to 07.00) and had free access to food and water. The animals were housed and tested in the same room. A radio, which was playing softly, provided background noise in the room. All testing was done between 09.00 and 17.00.

For the ex vivo studies, all experimental procedures were approved by the local ethical committees of Hasselt University (Ethische Commissie Dierproeven, ECD Hasselt) and Maastricht University (approval code: 2018-006; Licensed animal ethical committee Ministry of VWS, GZBIVVB981845) for animal experiments in accordance with governmental guidelines (Belgium: KB 29.05.13, The Netherlands: BWBR0003081, Wod; EU: 2010/63/EU). Two cohorts of twelve 2–3-month-old male C57BL/6 mice (Charles River, l’Arbresle, France) were used. The animals were housed socially in a standard open cage on sawdust bedding in an air-conditioned room (about 20 °C). They were kept under a reversed 12/12 h light/dark cycle (lights on from 19.00 to 07.00) and had free access to food and water.

All animal studies described were performed in concordance with the ARRIVE guidelines^24^. The experimenter was always blinded to the conditions during testing and data analysis. Treatment conditions were always balanced across the animals. All animals were euthanized after the experiment according to the European directive 2010/63/EU with 150 mg/kg pentobarbital (i.p.) unless otherwise specified.

### Materials

BAY-747 and runcaciguat were supplied by Bayer AG (Leverkusen, Germany)^25,26^. Donepezil, an acetylcholinesterase inhibitor (AChEI), was generously donated by Abbott (Weesp, The Netherlands). Methyl 2-hydroxyethyl cellulose (Tylose^®^ MH300) and Tween80 (polyoxyethylenesorbitan monooleate, cat# P8074) were purchased from Sigma-Aldrich Chemie bv (Steinheim, Germany). Forskolin was purchased from Tocris Bioscience (#1099, Abingdon, UK), rolipram was purchased from Abcam (#ab120029), and sulfo-NHS-SS-biotin (#A39258) and streptavidin-coated Dynabeads (#65601) were purchased from Thermo Scientific (Bleiswijk, The Netherlands).

Mouse anti-GluA1 was purchased from Merck Millipore (#MAB2263, Burlington, MA, USA). Rabbit anti-GluA1 phospho S845 was purchased from Abcam (#ab76321), and mouse anti-GAPDH was purchased from Fitzgerald Industries (#10R-G109A, Acton, MA, US) (all three antibodies successfully used previously^13,20^). Rabbit anti-TrkB (#4603S, Cell Signaling Technologies) and mouse anti-PSD95 (#56452, QED Bioscience) were a generous gift from Bayer (as used previously^27,28^).

### Treatment

BAY-747 and runcaciguat were freshly prepared on every experimental day and were dissolved in 0.5% Tylose solution (98% of the end volume) with 2% Tween80. Donepezil and L-NAME were also prepared fresh on every experimental day and were dissolved in saline.

For the long-term memory dose response curves, BAY-747 was tested at doses of 0.01, 0.03, 0.1, 0.3, 1.0 and 3.0 mg/kg. Runcaciguat was tested at doses of 0.01, 0.03, 0.1, 0.3, 1.0 and 3.0 mg/kg. Donepezil was tested at the active dose of 1.0 mg/kg. All compounds were tested in a time-dependent memory deficit model, i.e. a 24 h inter-trial interval OLT. The AChEI donepezil acted as a reference drug. BAY-747, runcaciguat and donepezil were administered p.o. (injection volume 2 ml/kg), 30 min before the learning trial (T1) of the OLT to investigate the effects on the memory acquisition process. The order of the treatments was balanced to prevent the data from being distorted by potential object- and side-preferences of the animals.

For the L-NAME experiments, L-NAME was administered at 30 mg/kg p.o. (injection volume 2 ml/kg) for 6 consecutive days. On days 5 and 6, behavioral testing was performed with an L-NAME induced memory deficit model in a 1 h inter-trial interval OLT. L-NAME was administered 1 h prior to the learning trial (T1) of the OLT. BAY-747 and runcaciguat were administered acutely on experimental days 5 and 6, 30 min before T1 at 0.3 mg/kg p.o. and 1.0 mg/kg p.o. (2 ml/kg) as based on the long-term memory dose–response curves. The order of the treatments was balanced to prevent the data from being distorted by potential object- and side-preferences of the animals.

For the in vivo memory acquisition plasticity measures, the dosing of BAY-747 and runcaciguat was chosen based on the difference in K_m_ factors for mouse and rats (6 and 3 respectively). Based on this, there was a potential for a shift of the dose response curve to the right of a single log-scale when converting doses from rats to mice (see^29^ for an explanation of calculation methods). Therefore, BAY-747 and runcaciguat were administered at 1.0 mg/kg p.o. (injection volume 4 ml/kg) to C57BL/6 mice based on the highest effective dose in Wistar rats for enhancing long-term memory. The treatments were administered 30 min before T1 in a 24 h inter-trial interval OLT. Due to the size of rat hippocampal slices, rats were not considered suitable for both these and the ex vivo experiments.

### Determination of plasma and brain exposure in rats

Rats were orally treated with BAY-747 (0.1 or 3 mg/kg) or runcaciguat (0.3 or 3 mg/kg) and 30 min after administration both blood and brain were collected after decapitation without prior anesthesia. The procedures for quantification of plasma and brain concentration of BAY‐747 and runcaciguat were performed as described previously^30^. In brief, after work-up and protein precipitation, the plasma concentrations in the samples were analyzed using an LC‐system (Kinetex 5 μm C18 100 A LC Column 150 × 4.6 mm) coupled to a 4500 Triple Quad Sciex mass analyzer (positive mode; Framingham, MA, USA) and a generic internal standard was added to the samples. A 5‐point calibration curve and quality control samples were used for relative quantification by high pressure liquid chromatography (HPLC)—tandem mass spectrometry (MS/MS). The brain-to plasma ratio (C_b_:C_p_) was calculated to determine the brain penetration of BAY-747 and runcaciguat in rats.

### Tissue distribution by quantitative whole-body autoradiography

The procedures were performed as described previously^30^. In brief, [^14^C]BAY-747 was administered orally at 1 mg/kg and [^14^C] runcaciguat was administered orally at 3 mg/kg (equivalent to a radioactive dose of 4.8 MBq/kg and 7.2 MBq/kg respectively) to male Wistar rats. The animals were subsequently sacrificed at 2, 4, 8, 24, 72 and 168, one animal per time point, by cutting the carotid artery while under deep isoflurane anesthesia (approx. 2.5% v/v). Blood and plasma were collected from the tail vein appr. 3–5 min before animals were sacrificed. Carcasses were deep frozen in solid carbon dioxide/dichloromethane (− 70 °C) and embedded in a block of aqueous carboxymethylcellulose gel (50 g CMC/L) and frozen again in solid carbon dioxide/heptane (− 70 °C). Sagittal whole-body sections were cut (50 μm thickness) at − 25 °C, freeze-dried and exposed to FUJI phosphor imaging plates for 1–4 days. Radioactivity distribution in the whole-body sections was detected by radioluminography technique using FUJI BAS-5000. Blood standards spiked with [^14^C] (up to 10 preassigned concentrations measured by LSC exposed together with each animal) were used as calibrators for quantitation of radioactivity in tissues and organs. The electronic images were analyzed using the validated BAS Reader scan software. Concentrations of radioactivity were expressed as µg equivalents of BAY-747 or runcaciguat per L tissue (µg-eq/L). The upper and lower limits of quantification by quantitative whole-body autoradiography were assessed based on calibrated blood standards.

### Object location testing

The Object Location Task (OLT) was derived from the Object Recognition Test (ORT)^31^. The OLT was performed as previously described for rats^32,33^ and mice^20,34^. In brief, the apparatus consisted of a circular arena with an 83 cm diameter for rats and 40 cm diameter for mice, and 40 cm high walls (for both rats and mice), made of polyvinyl chloride. The front half of the arena was transparent, while the back-half was untransparent and gray in color. Fluorescent red tubes and a light bulb provided a constant, equal illumination of approx. 20 lx on the floor of the arena.

A testing session consisted of two trials of 3 min (rats) or 4 min (mice) each. The first trial was the learning trial (T1), in which there were two identical objects placed symmetrically on the mid-line of the arena, about 10 cm (rats) or 5 cm (mice) from the nearest side-wall. In the second test trial (T2), one of the objects was moved to a new location, approx. 20 cm (rats) or 10 cm (mice) forward or backward from the original position. For rats, there were four sets of objects, consisting of (1) a brown 1 L glass bottle filled with water (10 cm diameter and 22 cm height); (2) a solid aluminum cube with a square bottom and tapering top (13 × 8 × 8 cm); (3) a white cube with rounded edges and a smaller, cylindric shaped bottom stand (13 × 8 × 8 cm); (4) a solid metal cube (10 × 5 × 7.5 cm) with two holes (1.9 cm diameter). The rats were unable to displace the objects. For mice, two sets of objects were used: (1) a solid aluminum cube with a square bottom and tapering top (4.5 × 4.5 × 8.5 cm); (2) a solid metal cube (2.5 × 5 × 7.5 cm) with two holes (1.5 cm diameter). The mice were unable to displace the objects.

After the T1, the rodent is placed back in its home cage for a specific time interval, before being allowed to explore during T2. For measuring long-term memory, a 24 h interval between T1 and T2 was used to measure natural forgetting. Hence, under vehicle conditions, memory performance will be low. For the L-NAME experiments, short-term memory impairments were measured using a 1 h inter-trial interval. Under vehicle conditions, memory performance will be high. The total time the rodents spent exploring each object during T1 and T2 was recorded manually on a personal computer by an experimenter blinded to the treatment conditions. Of note, for the specific plasticity experiments with mice in this study, the animals were sacrificed at the timepoint of T2 testing, i.e. 24 h after T1. Thus, a test trial was not performed (based on procedures previously described^20^).

#### Object location task procedures

Exploration was defined as the animal directing the nose to the object (< 2 cm distance) or touching the object with the nose, while sitting on the object was not scored as exploratory behavior. Animals with total exploration times < 7 s during T1 or < 9 s during T2 were excluded from the analysis, since a minimal amount of object interaction is needed for reliable object discrimination measures^35^. The objects and the arena were always cleaned with 70% ethanol between trials, and both the object type and location were used in a balanced manner.

The first 2 weeks, animals were handled daily and habituated thoroughly to the arena, the objects, and the experimental procedures. The animals were allowed to explore the empty arena twice, and were then allowed to explore the arena with two objects for two days. The animals were subsequently familiarized with the testing procedure until discrimination performance was stable. For the long-term memory experiments, vehicle testing was done with 24 animals and all other conditions were tested in 16 animals (within design). For the L-NAME experiments, the vehicle and vehicle + L-NAME conditions were performed in 24 animals, while the BAY-747 and runcaciguat conditions were tested in 12 animals. The in vivo plasticity experiments were performed with 8 animals per treatment group (between design). The experimenter was always blinded to the conditions being tested both during test procedures and during data analysis. Treatment conditions were always assigned in a balanced manner.

The time spent exploring the objects during T1 and T2 was recorded on a personal computer. The time spent exploring the two identical objects during T1 will be represented as ‘Object_1’ and ‘Object_2’, while during T2 the time spent exploring the familiar object is represented as ‘Familiar’ and the novel object is represented as ‘Novel’. This means that the total exploration time during T1 can be defined as e1 = Object_1 + Object_2, whereas the total exploration time during T2 can be defined as e2 = Familiar + Novel. The discrimination index d2, as a representative for memory performance, is then be calculated by d2 = (Novel − Familiar)/e2. In this way, the d2 measures the difference in exploration times between the novel and the old position of the object, while correcting for total exploration times, making it a relative measure of memory performance even if exploration times would be affected by the treatment.

### Chemical LTP protocol

The chemical LTP protocol was based on previous literature which showed synaptic potentiation in acute slices after treatment with forskolin and rolipram^36^. Untreated wild-type C57BL/6 mice were sacrificed by cervical dislocation without prior anesthesia, and both hippocampi were quickly isolated on ice. 400 µm thick coronal hippocampal slices were obtained using a McIlwain tissue chopper and the slices were transferred to a recovery chamber containing artificial cerebrospinal fluid (ACSF; 124 mM NaCl, 25 mM NaHCO_3_, 10 mM glucose 4.4 mM KCl, 2 mM CaCl, 2 mM MgSO_4_, and 1 mM Na_2_HPO_4_) under constant oxygenation with 95% O_2_ and 5% CO_2_ at 37 °C. The slices were allowed to recover for three hours, and were transferred to a modified 6-well plate (costar) containing individual incubation chambers in order to achieve simultaneous incubation of all experimental groups. The incubation chambers always contained ACSF, with or without treatments, kept at 37 °C under constant oxygenation. To mimic memory acquisition-like processes, a BAY-747/runcaciguat incubation protocol was performed prior to the chemical LTP (cLTP) protocol as follows: 10 min of incubation with BAY-747/runcaciguat or vehicle (0.1% DMSO), 10 min rest in ACSF, 15 min cLTP protocol or vehicle (0.3% DMSO) and 15 min rest in ACSF before processing and collection of the slices. Please see Supplemental Methods Figs. M1–M3 for an overview of the custom cLTP set-up.

For the incubation with BAY-747 or runcaciguat, the slices were incubated with the optimum concentration of BAY-747 or runcaciguat (pre-diluted in DMSO) for 10 min, diluted in ACSF (final concentration 0.1% DMSO), with 0.1% DMSO as the vehicle condition. After the 10 min rest in ACSF, a cLTP procedure was performed to induce a weak stimulation similar to a weak memory stimulus in vivo. To induce this weak cLTP, the hippocampal slices were incubated with 50 µM forskolin and 0.1 µM rolipram (pre-diluted in DMSO) for 15 min in ACSF under constant oxygenation with a final concentration of 0.3% DMSO. The vehicle condition therefore also contained 0.3% DMSO. After cLTP induction, the slices were allowed to recover for 15 min in fresh ACSF before collection in a 24-well plate containing ice-cold ACSF. The optimum concentration of BAY-747 and runcaciguat was determined based on a concentration–response curve in combination with weak stimulation: 1–10-100 nM. For the experiments with the optimum concentrations, 4 different conditions were used as described in Table 1.

**Table 1:** Incubation conditions during the BAY-747 and runcaciguat ex vivo cLTP experiments.

Experimental condition	Treatment during 10 min BAY-747 incubation	Treatment during cLTP protocol
Vehicle	0.1% DMSO	0.3% DMSO
BAY-747 only (without WS)	100 nM BAY-747	0.3% DMSO
BAY-747 + WS	100 nM BAY-747	50 µM forskolin + 0.1 µM rolipram
Weak stimulation (WS) only	0.1% DMSO	50 µM forskolin + 0.1 µM rolipram

Experimental condition	Treatment during 10 min runcaciguat incubation	Treatment during cLTP protocol
Vehicle	0.1% DMSO	0.3% DMSO
Runcaciguat only (without WS)	10 nM runcaciguat	0.3% DMSO
Runcaciguat + WS	10 nM runcaciguat	50 µM forskolin + 0.1 µM rolipram
Weak stimulation (WS) only	0.1% DMSO	50 µM forskolin + 0.1 µM rolipram

Described are the experimental conditions and corresponding exposures during the BAY-747 and runcaciguat incubation period and the chemical LTP protocol. WS: weak stimulation induced by 50 µM forskolin and 0.1 µM rolipram.

### Sample processing for western blotting

For the in vivo plasticity experiments, the animals were sacrificed 24 h after the T1 of the OLT by cervical dislocation without prior anesthesia. The hippocampi were quickly isolated and 400 µm thick coronal slices were obtained with a McIlwain tissue chopper. The slices were then collected in a 24-well plate (one well per mouse) containing ice-cold ACSF.

The processing procedures for the ex-vivo cLTP and in vivo plasticity experiments were identical after the point of collection in 24-well plates containing ice-cold ACSF. The slices were incubated with 1 mM sulfo-NHS-SS-biotin for 1 h on ice. Slices were subsequently quenched with 100 mM glycine to remove any excess biotin, washed with ice-cold ACSF, collected in tubes and snap frozen in liquid nitrogen. Frozen hippocampal slices were mechanically dissociated in lysis buffer (1 mM EDTA, 1 mM EGTA, 1% glycerol, 1% igepal CA-630, 0.1% Triton-X100, and phosphatase and protease inhibitors in PBS). Protein concentrations were determined using a Lowry protein assay (Bio-Rad Laboratories, Veenendaal, The Netherlands). To obtain membrane fractions, 30 µg of protein lysate was incubated overnight with 25 µl of streptavidin-coated dynabeads at 4 °C under constant rotation. The membrane fractions bound to the dynabeads were separated from cytosolic proteins using magnetic precipitation, and were eluded from the beads using 1 × sample buffer (1 M Tris–HCl, 75% glycerol, 15% β-mercaptoethanol, 6% SDS, and 0.025% brome phenol blue in MilliQ) and incubation at 100 °C for 5 min. Total protein fractions were also processed using 1 × sample buffer and incubation at 100 °C for 5 min.

### Western blotting

Protein lysates (both surface and total fractions) were resolved in SDS-PAGE For GluA1, PSD95, and TrkB, 7.5% polyacrylamide gels were used, whereas for BDNF, a 14% gel was used. The separated proteins were transferred onto nitrocellulose membranes and blocked with odyssey blocking buffer diluted 1:1 in PBS (Li-Cor, Lincoln, NE, USA) for 1 h at room temperature (for BDNF, TBS was used for the entire protocol). Primary antibody incubation was done overnight at 4 °C using the following antibodies: 1:2000 mouse anti-GluA1, 1:2000 rabbit anti-pS845 GluA1, 1:1000 rabbit anti-BDNF, 1:250 rabbit anti TrkB, 1:2000 mouse anti-PSD95, and 1:2,000,000 mouse anti-GAPDH (Fitzgerald Industries). Membranes were subsequently washed with PBS/PBS-0.1% tween-20 (PBS-T)/PBS for 10 min each and incubated with secondary antibodies donkey anti-mouse IRDye 680 and goat anti-rabbit IRDye 800 (both 1:10,000, Li-Cor) for 1 h at room temperature. After washing with PBS/PBS-T/PBS, the membranes were dried, and bands were visualized using an Odyssey CLx Infrared Imaging System (Li-Cor). Protein bands were quantified using ImageJ. For all western blots containing both membrane and total protein fractions, the same reference protein sample with a known protein concentration was loaded to control for differences in signal intensity, background intensity, and scanning intensity between the different blots. Raw intensity measures were also normalized to GAPDH to control for loading differences. Technical outliers were excluded (i.e. background artifacts, abnormal bands, unresponsive slices in the cLTP). The data were analyzed by using one-way ANOVA followed up by post-hoc LSD comparisons.

### Statistical analysis

All data were analyzed using a one-way ANOVA followed up by post-hoc LSD comparisons to compare the different treatment conditions. For the behavioral experiments, an independent-samples *t* test was performed to compare the treatment conditions to a virtual zero with SEM = 0.065 as a representation of chance level, based on historical data^37^. Significance was set as α = 0.05. For the in vivo synaptic plasticity measures, differential effects of sGC stimulators and activators could potentially be expected which could confound the results of a one-way ANOVA. Therefore, independent-samples *t* tests to vehicle were performed instead.

## Results

### BAY-747 and runcaciguat enhance long-term memory acquisition processes

**Figure 1:**
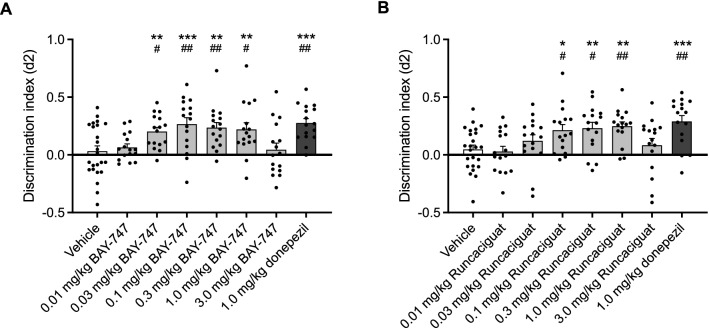
Effects of different doses of BAY-747, runcaciguat and donepezil on long-term memory acquisition. (**A**) Treatment with 0.03–1.0 mg/kg BAY-747 enhanced memory performance both compared to virtual zero (independent samples *t* test) and to vehicle (one-way ANOVA with post-hoc LSD tests). Similarly, the positive control donepezil enhanced memory performance at 1.0 mg/kg. Both BAY-747 and donepezil were administered p.o. (2 ml/kg) at 30 min before T1 in a 24 h interval OLT. (**B**) Treatment with 0.1–1.0 mg/kg runcaciguat enhanced memory performance both compared to virtual zero and to vehicle, as did the positive control and reference compound donepezil at 1.0 mg/kg. Both runcaciguat and donepezil were administered 30 min before T1 in a 24 h interval OLT to enhance memory acquisition processes. Data are represented as mean + SEM. A difference from virtual zero (0 ± 0.065) is depicted with hashes (independent samples *t* tests, ^#^P < 0.05; ^##^P < 0.01). A difference from the vehicle condition is depicted with asterisks (one-way ANOVA, LSD *t* tests, *P < 0.05; **P < 0.01; ***P < 0.001).

For BAY-747, a one-way ANOVA revealed differences between exploration times in both T1 (e1: F (7, 128) = 5.12; P < 0.001) and T2 (e2: F (7,128) = 3.03; P < 0.01). Since all exploration times remained appropriately high for reliably measuring memory formation (e1 > 7 s and e2 > 9 s), a detailed overview of the exploration times and analysis can be found in Supplemental Table S1. Memory performance was significantly higher than virtual zero in animals that received 0.03, 0.1, 0.3 and 1.0 mg/kg BAY-747 and 1.0 mg/kg donepezil, as indicated by independent-samples *t* tests. No significant difference from virtual zero was detected for 0.01 and 3.0 mg/kg BAY-747. A one-way ANOVA revealed significant differences for the discrimination index d2 between treatment conditions (d2: F (7, 128) = 4.92, P < 0.001). Post-hoc LSD *t* tests revealed that administration of 0.03, 0.1, 0.3 and 1.0 mg/kg BAY-747 30 min before T1 resulted in a significantly higher long-term memory performance compared to the vehicle condition (Fig. 1A). Similar results were found for the positive control donepezil at 1.0 mg/kg.

For runcaciguat, a one-way ANOVA revealed no differences between exploration times in neither T1 (e1: F (7, 128) = 0.63; n.s.) nor T2 (e2: F (7,128) = 1.80; n.s.). The discrimination index (d2) as a measure for memory performance was significantly higher than virtual zero in animals that received 0.1, 0.3 and 1.0 mg/kg runcaciguat and 1.0 mg/kg donepezil, as indicated by independent-samples *t* tests. No significant difference from virtual zero was detected in any of the other runcaciguat conditions. A one-way ANOVA revealed significant differences for the discrimination index d2 between treatment conditions (d2: F (7, 128) = 4.39, P < 0.001). Post-hoc LSD *t* tests revealed that treatment with 0.1, 0.3 and 1.0 mg/kg runcaciguat administered 30 min before T1 resulted in significantly higher memory performance when compared to the vehicle condition (Fig. 1B). Once again, the positive control and reference compound donepezil also enhanced long-term memory performance.

### BAY-747 attenuates L-NAME induced short-term memory impairments, whereas runcaciguat does not

**Figure 2:**
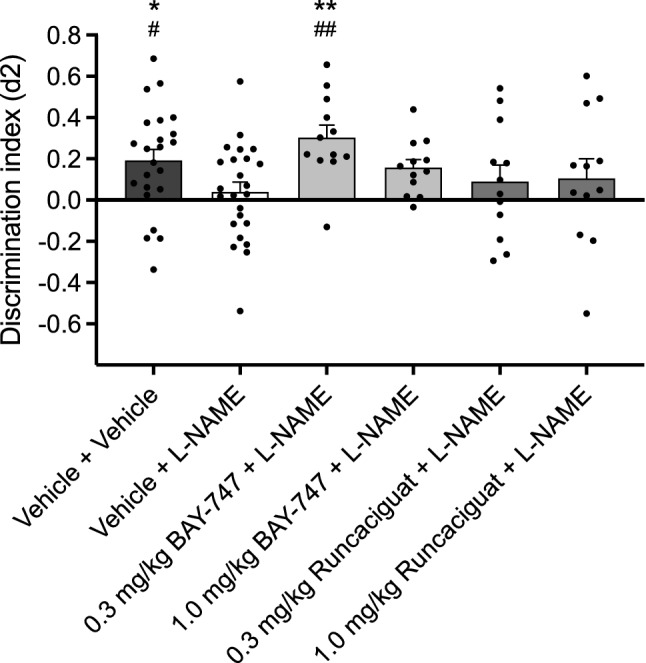
The effects of BAY-747 and runcaciguat on L-NAME induced short-term memory impairments. Vehicle animals showed healthy memory performance which was significantly higher than virtual zero (independent-samples *t* test), whereas L-NAME treated animals showed poor memory performance which was significantly lower than vehicle treated animals (independent-samples *t* test) and not significant from zero. Treatment with 0.3 mg/kg BAY-747 reversed L-NAME induced memory impairments since the discrimination index was both significantly higher compared to virtual zero and L-NAME treated animals (one-way ANOVA). Treatment with 1.0 mg/kg BAY-747 did not alter short-term memory performance after L-NAME treatment. Runcaciguat was unable to enhance memory performance under L-NAME treated conditions in the doses tested. Both BAY-747 and runcaciguat were administered p.o. at 2 ml/kg 30 min before T1 in a 1 h interval OLT. L-NAME was administered for 6 consecutive days at 30 mg/kg p.o. and administered 1 h before T1 on testing days. Hashes represent a significant difference from virtual zero (0 ± 0.05) as measured with independent-sample *t* tests, ^##^P < 0.01; ^###^P < 0.001. Asterisks represent a significant difference from L-NAME, *P < 0.05; **P < 0.01. Data are represented as mean + SEM.

No differences in exploration times during T1 were found for either BAY-747 (one-way ANOVA e1: F(2, 45) = 1.307; n.s.) or runcaciguat (one-way ANOVA e1: F(2, 45) = 0.068; n.s.) and similarly, no differences in exploration times were found during T2 (one-way ANOVA e2; BAY-747: F(2, 45) = 0.950; n.s.; runcaciguat: F(2, 45) = 1.563; n.s.). For a detailed overview of the exploration times, see Supplementary Table S2. Vehicle-treated animals showed a healthy short-term memory performance that was significantly better than virtual zero (independent-samples *t* test). 6-Day consecutive L-NAME treatment (30 mg/kg p.o.) impaired short-term memory performance, since the discrimination index d2 was not significant from virtual zero, while significantly lower than vehicle (independent-samples *t* test). A one-way ANOVA revealed a treatment effect of BAY-747 on L-NAME induced memory impairments (F(2, 45) = 6.587; P < 0.01) and post-hoc LSD tests showed that animals treated with L-NAME in combination with 1.0 mg/kg BAY-747 had significantly higher short-term memory performance compared to both virtual zero and L-NAME only, whereas animals treated with L-NAME in combination with 0.3 mg/kg BAY-747 did not perform better compared to both virtual zero and vehicle. Contrary to BAY-747, no treatment effect of runcaciguat on L-NAME induced short-term memory performance could be identified (F(2, 45) = 0.294; n.s.; Fig. 2).

### BAY-747 and runcaciguat enhance AMPA receptor dynamics in an ex vivo acquisition-like cLTP model

**Figure 3:**
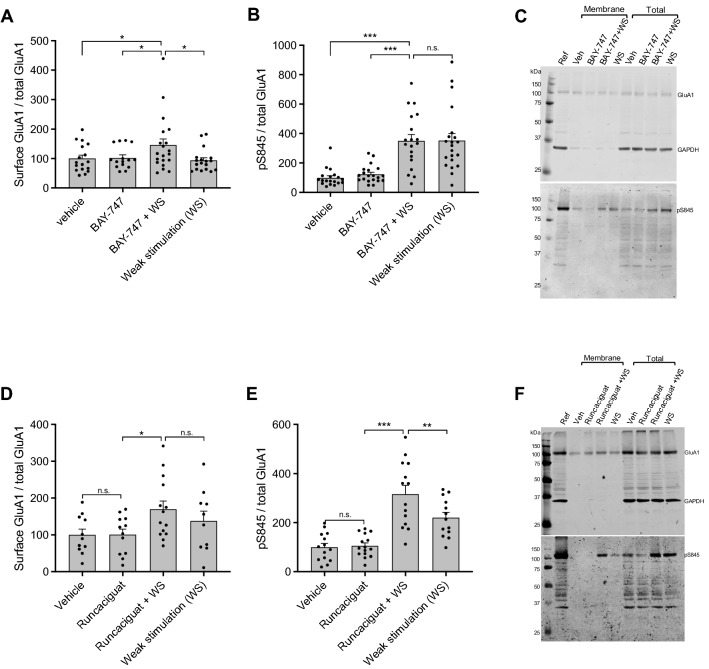
The effects of BAY-747 and runcaciguat on GluA1-containing AMPAR mobilization to the surface in an ex-vivo acquisition-like cLTP model. (**A**) Incubation with 100 nM BAY-747 in combination with WS enhanced GluA1-containing AMPAR mobilization as measured by the surface GluA1/total GluA1 ratio, compared to vehicle, 100 nM BAY-747 only, and WS only. (**B**) The phosphorylation levels of S845 on GluA1 were enhanced by WS and a combination of BAY-747 and WS, yet BAY-747 could not further enhance S845 phosphorylation in combination with WS when compared to WS only. (**C**) Representative image of a full BAY-747 western blot. (**D**) Incubation with 10 nM runcaciguat in combination with WS partially enhanced GluA1-containing AMPAR mobilization to the surface as measured by the surface GluA1/total GluA1 ratio, since the mobilization measure was significantly higher than vehicle and runcaciguat only, but not to WS only while WS in itself did not affect AMPAR mobilization. (**E**) The phosphorylation levels of S845 on GluA1 were enhanced by the combination of runcaciguat and WS when compared to WS only, vehicle, and runcaciguat only. (**F**) representative image of a full western blot. Each western blot contained an identical loading control with a known protein concentration as an internal correction for signal intensity/background differences between blots. Since GAPDH barely associates with membrane proteins, the membrane protein fraction contained no or very little GAPDH on the western blot. Only pS845 of the total protein samples was analyzed. BAY-747 was incubated at a concentration of 100 nM, while runcaciguat was incubated at a concentration of 10 nM. Data are represented as mean + SEM. n = 15–23. *P < 0.05; **P < 0.01; ***P < 0.001 as measured with post-hoc LSD tests. For scans of all western blots analyzed, please see Supplemental WB Figs. A1–A3 for BAY-747 and Supplemental WB Figs. B1–B5 for Runcaciguat.

A concentration–response curve experiment with BAY-747 using 1, 10 and 100 nM in combination with weak stimulation (WS) identified 100 nM BAY-747 as the optimum concentration for enhancing AMPAR mobilization to the surface (see Supplemental Fig. S1). A one-way ANOVA revealed an effect of the experimental condition on AMPAR mobilization as represented by the surface GluA1/total GluA1 ratio (F(3, 66) = 2.867; P < 0.05). Post-hoc LSD tests further identified that the combination of 100 nM BAY-747 and WS enhanced AMPAR mobilization to the surface compared to vehicle, BAY-747 alone, and WS alone Fig. 3A). No effects of the experimental conditions could be identified for the amount of surface GluA1-containing AMPARs (F(3, 86) = 0.468; n.s.;) or the total amount of GluA1 (F(3, 85) = 0.791; n.s.; see Supplemental Fig. S2). The phosphorylation of Ser845 (pS845) on GluA1 was found to be affected by the experimental conditions (F(3, 75) = 16.89; P < 0.001) and post-hoc LSD tests revealed that both WS alone and WS in combination with BAY-747 treatment enhanced pS845-GluA1 compared to vehicle and BAY-747 alone. Yet no differences could be found between WS and BAY-747 + WS (Fig. 3B,C).

A concentration–response curve with runcaciguat using 1, 10 and 100 nM in combination with WS identified 10 nM runcaciguat as the optimum concentration (see Supplemental Fig. S1). A one-way ANOVA revealed an effect of the experimental conditions on AMPAR mobilization (F(3, 42) = 0.912; P < 0.05) and post-hoc LSD tests identified that 10 nM runcaciguat in combination with WS significantly enhanced GluA1-containing AMPAR mobilization to the surface compared to vehicle and runcaciguat only. No difference was found in comparison with WS only, yet WS in itself did not enhance AMPAR mobilization, which is suggestive of an intermediate effect of runcaciguat in combination with WS on AMPAR trafficking (Fig. 3D). No effects were found on surface GluA1 (F(3, 53) = 1.168; n.s.) or total GluA1 (F(3, 48) = 1.168; n.s.; see Supplemental Fig. S2). S845 phosphorylation was affected by the experimental conditions as shown with a one-way ANOVA (F(3, 52) = 20.91; P < 0.001) and post-hoc LSD tests revealed that 10 nM runcaciguat in combination with WS enhanced pS845-GluA1 compared to vehicle, runcaciguat alone, and WS alone (Fig. 3E,F).

### Brain penetrating properties of BAY-747 and runcaciguat

For the interpretation of the obtained in vivo data and for the potential mode of action of BAY-747 and runcaciguat, blood–brain penetration is a critical parameter. We analyzed the blood–brain penetration by whole-body autoradiography and by direct measurement of BAY-747 and runcaciguat in both in plasma and brain (see Table 2). Based on LC–MS/MS analyses, BAY-747 showed a brain to plasma ratio of 0.6 ± 2.0 (gMean; gSD) at the investigated time frame, reflecting a relatively high brain penetration of 60%. Runcaciguat showed a brain to plasma ratio of 0.1 ± 1.4 in rats (gMean, gSD), which reflects a brain penetration of about 10%. These data are well in line with those obtained from whole-body autoradiography using ^14^C-labelled compounds, where a brain-to-blood ratio of 0.6 for BAY-747 and 0.2 for runcaciguat was obtained based on AUC. Taken the blood-to-plasma ratio for runcaciguat (0.679) and BAY-747 (1.59) into consideration, the brain-to-plasma ratio for runcaciguat is 0.13 and for BAY-747 is 0.38 based on the data from autoradiography.

**Table 2:** Brain penetrating properties of BAY-747 and runcaciguat.

	LC–MS/MS analyses		Whole-body autoradiography (based on AUC)	
BAY-747	Brain:plasma ratio	0.60 (2.0)	Brain:plasma ratio	0.38
			Brain:blood ratio	0.60
			Blood:plasma ratio	1.59
Runcaciguat	Brain:plasma ratio	0.10 (1.40)	Brain:plasma ratio	0.13
			Brain:blood ratio	0.20
			Blood:plasma ratio	0.68

Described is the brain:plasma ratio of BAY-747 and runcaciguat measured with LC–MS/MS [displayed as gMean (gSD)] and measured with whole-body autoradiography. A brain:plasma ratio > 0.04 is generally considered as good brain penetration. The blood to plasma ratio is determined to calculate from blood to plasma partitioning of the compounds.

### BAY-747 and runcaciguat enhance in vivo hippocampal plasticity associated with memory acquisition via differential mechanisms

**Figure 4:**
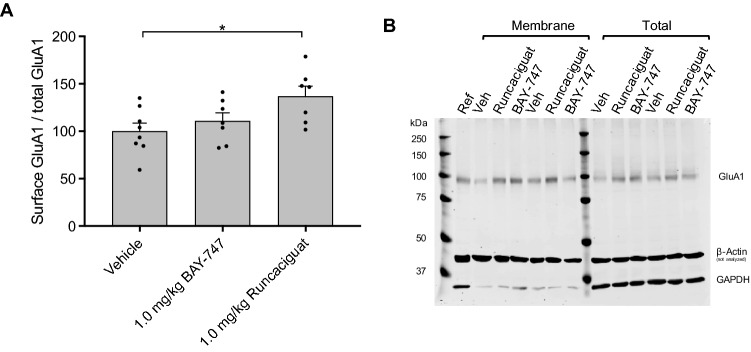
The effects of BAY-747 and runcaciguat on GluA1-containing AMPAR trafficking in memory acquisition processes in vivo. (**A**) 1.0 mg/kg runcaciguat (p.o. 4 ml/kg) administered 30 min before the learning trial of an OLT, enhanced GluA1-containing AMPAR trafficking to the surface measured 24 h after learning. In contrast, BAY-747 did not enhance GluA1 trafficking. (**B**) Representative full western blot image. β-actin was stained in case GAPDH had run off the 7.5% gel. GAPDH was used for normalization of raw values, since β-actin bands were saturated on the membrane. Asterisks represent statistical significance compared with vehicle as measured with independent-samples *t* tests. *P < 0.05. n = 8; data are represented as mean + SEM. For scans of all western blots analyzed, please see Supplemental WB Fig. C1.

The effects of BAY-747 and runcaciguat on plasticity markers in the hippocampus was measured during memory acquisition processes in vivo. Administration of 1.0 mg/kg runcaciguat p.o. enhanced GluA1-containing AMPAR trafficking in the hippocampus as reflected by an increased surface GluA1/total GluA1 ratio measured 24 h after learning (Fig. 4A,B). Surprisingly, BAY-747 did not affect GluA1-containing AMPAR dynamics in the hippocampus. Neither runcaciguat nor BAY-747 treatment showed an effect after 24 h at the surface GluA1 levels or total GluA1 levels (see Supplemental Fig. S3).

**Figure 5:**
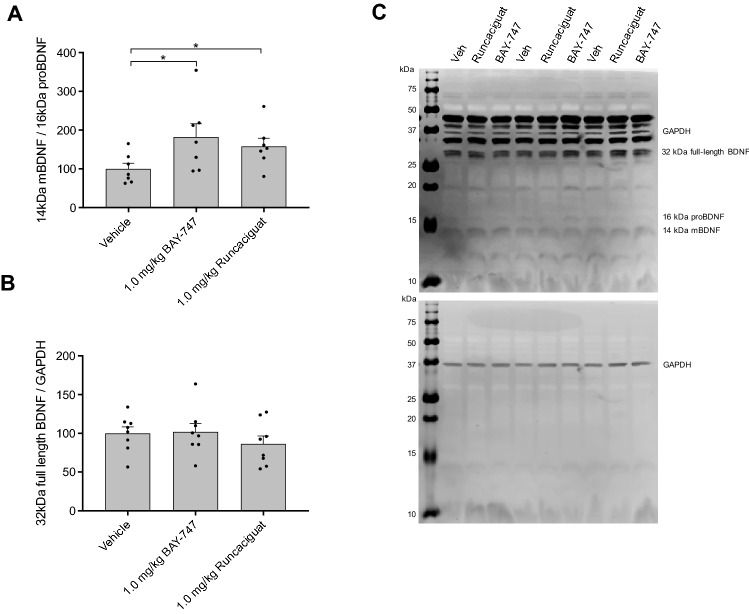
The effects of BAY-747 and runcaciguat on BDNF dynamics in memory acquisition processes in vivo. (**A**) 1.0 mg/kg BAY-747 and 1.0 mg/kg runcaciguat treatment (p.o. 4 ml/kg) enhanced 14 kDa mBDNF levels compared to 16 kDa proBDNF. (**B**) The amount of the 32 kDa full length BDNF fragment was not affected, suggesting a shift in cleavage toward more 14 kDa mBDNF upon BAY-747 or runcaciguat treatment. (**C**) Representative western blot image. Of note, different scanning intensities were used to analyze the different bands to prevent loss of signal or the analysis of saturated signals. Compounds were administered 30 min before the learning trial of an OLT, and the protein levels were measured 24 h after learning. Asterisks represent a significance from vehicle as measured with independent-samples *t* tests: *P < 0.05. n = 7–8; data are represented as mean + SEM. For scans of all western blots analyzed, please see Supplemental WB Fig. C2.

24 h after treatment, both 1.0 mg/kg BAY-747 and 1.0 mg/kg runcaciguat treatment increased the 14 kDa mBDNF/16 kDa proBDNF ratio (Fig. 5A) indicative of relatively increased mBDNF levels in comparison to 16 kDa proBDNF. The protein levels of full-length unspliced 32 kDa BDNF remained unaltered after BAY-747 or runcaciguat treatment (Fig. 5B,C).

**Figure 6:**
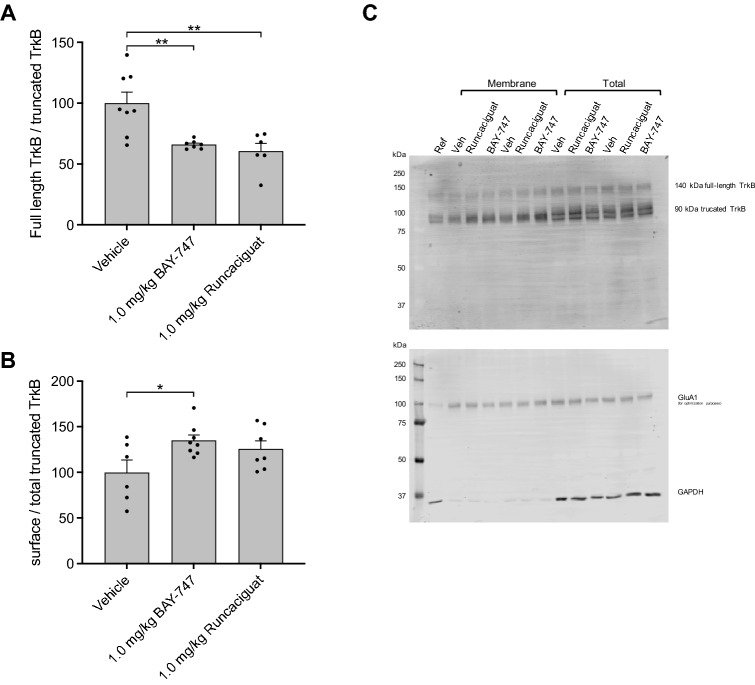
The effects of BAY-747 and runcaciguat on TrkB dynamics in memory acquisition processes in vivo. (**A**) Both 1.0 mg/kg BAY-747 and 1.0 mg/kg runcaciguat treatment resulted in a higher presence of truncated TrkB measured at the cell surface compared to full-length TrkB. (**B**) BAY-747 specifically enhanced the mobilization of truncated TrkB to the surface. (**C**) Representative image of a western blot. GluA1 was co-stained for optimization purposes of the protocol and was not analyzed in this western blot due to background artifacts in the IR700 channel. Asterisks represent a significant difference from vehicle as measured with independent-samples *t* tests: *P < 0.05; **P < 0.01. n = 7–8; data are represented as mean + SEM. For scans of all western blots analyzed, please see Supplemental WB Fig. C3.

When measuring TrkB protein levels, 1.0 mg/kg BAY-747 and runcaciguat decreased the ratio between truncated (90 kDa) TrkB/full length (140 kDa) TrkB in the membrane protein fraction (Fig. 6A), indicating there was relatively more truncated TrkB present on the cell surface compared to full-length TrkB. However, when focusing on the mobilization of TrkB, only BAY-747 enhanced the mobilization of truncated TrkB to the surface (Fig. 6B,C). Full-length TrkB remained unaffected by BAY-747 or runcaciguat treatment (see Supplemental Fig. S4).

## Discussion

### BAY-747 and runcaciguat enhance long-term memory acquisition processes in the OLT

Both BAY-747 and runcaciguat were found to enhance long-term memory in rats, when administered p.o. 30 min before the learning trial of a 24 h interval OLT, thereby affecting the memory acquisition time-window^38^. BAY-747 fully enhanced memory acquisition processes in a dose-range between 0.03 mg/kg p.o. and 1.0 mg/kg p.o., whereas the therapeutic window of runcaciguat ranged from 0.1 mg/kg p.o. to 1.0 mg/kg p.o. 1.0 mg/kg donepezil p.o. was used as a positive control in this paradigm and also fully enhanced memory acquisition processes. The memory acquisition enhancing properties of BAY-747 and runcaciguat are in concordance with previous research using sGC stimulators^13,15,39^.

### BAY-747 and runcaciguat differentially affect subchronic L-NAME induced short-term memory impairments

In order to investigate the effects of BAY-747 and runcaciguat on spatial memory in the OLT in a model for upstream NOS/NO deficiencies, rats were subchronically treated with 30 mg/kg L-NAME p.o. for 6 consecutive days, which previously was found to induce short-term memory impairments^40^. Rats were tested at day 5 and day 6 of L-NAME treatment, and BAY-747 and runcaciguat were administered 30 min before T1 of a 1 h interval OLT. Subchronic L-NAME treatment successfully induced short-term memory impairments, and the sGC stimulator BAY-747 fully reversed this at a dose of 0.3 mg/kg p.o. Interestingly, the sGC activator runcaciguat did not reverse L-NAME induced memory impairments at 0.3 mg/kg and 1.0 mg/kg p.o., despite the effectiveness of these doses in the long-term memory paradigm. L-NAME is a brain-penetrant inhibitor of cNOS^41,42^, and in 1990, Mülsch and Busse^43^ already showed that L-NNA, another NOS inhibitor, was able to inhibit NO release and antagonize sGC in endothelial cells. Additionally, L-NAME treatment has been shown to inhibit the enhanced fear memory learning induced by YC-1, an sGC activator/stimulator^44^. A strong reduction in NO and basal NO-induced sGC activity could explain why sGC stimulation still reverses L-NAME induced memory impairments as Fe(II)sGC is not affected by L-NAME treatment. Thus the stimulator would take over the role of endogenous NO and restore normal NO–sGC–cGMP functioning, thereby reversing memory impairments induced by NO deficiencies. The sGC activator, acting only on Fe(III)sGC and apo-sGC, may not boost cGMP production enough under too low physiological Fe(III)sGC/apo-sGC levels to account for the reduced cGMP production due to NO deficiencies. Indeed, under physiological conditions, treatment with an sGC activator only accounts for a small amount of sGC activity compared to NO^16^. However, under high NO conditions, Fe(II)sGC is known to be degraded, hence sGC signalling can be functionally recovered in a mechanism-based manner by apo-sGC activators that re-establish cGMP formation^45^.

Nonetheless, the mechanisms of L-NAME are complex. For example, L-NAME can have hypertensive effects, especially after (sub)chronic treatment, yet in multiple studies using Wistar rats, daily administration of L-NAME at doses similar to or higher than the current study only induced hypertensive effects after more than 1 week of chronic treatment^46–49^. Since our subchronic treatment window was smaller than 1 week (6 days), only a marginal contribution of hypertensive effects to the L-NAME memory impairment model could be expected. Nevertheless, previous literature points at potential differential effects of L-NAME-induced NOS inhibition on sGC stimulator and activator actions. In pulmonary and systemic arterial beds, the vasodilatory response of sGC stimulator BAY41-8543 was markedly reduced by L-NAME treatment, whereas the vasodilatory response of sGC activator BAY60-2770 was increased by L-NAME^50^. Interestingly, these effects of L-NAME on sGC activators may be strongly related to the type of cNOS, as the effects of sGC activator BAY41-2272 on corpus carvenosum muscle relaxation were similarly repressed by L-NAME treatment in neuronal (n)NOS knock-out mice as in wild-type mice, whereas in endothelial (e)NOS knock-out mice the BAY41-2272 responses were unaffected by L-NAME^51^. Although most studies are investigating sGC stimulators and activators both alone and in combination with L-NAME focus on the cardiovascular system with mostly eNOS-derived NO, it may be suggested that L-NAME could exert different effects on sGC stimulators and activators in tissues where NO is mostly derived from nNOS production, e.g. the central nervous system. Given the brain-penetrant properties of L-NAME, a non-penetrant cNOS inhibitor could be used in future research to further elucidate the mechanisms behind the different effects of brain-penetrant sGC stimulators and activators.

### BAY-747 and runcaciguat enhance GluA1-containing AMPAR mobilization to the surface ex vivo, yet exert differential hippocampal plasticity mechanisms in vivo

To investigate the mechanisms behind enhancing memory acquisition processes, BAY-747 and runcaciguat were tested in an *ex-vivo* cLTP model to mimic memory acquisition processes in hippocampal mouse slices. This model was also previously used to test the mechanistic potential of sGC stimulator vericiguat on hippocampal neuronal plasticity in the absence of a blood–brain barrier limitation^13^, and therefore allows for a direct comparison. Both the sGC stimulator BAY-747 and the sGC activator runcaciguat were able to enhance the mobilization of GluA1-containing AMPARs to the surface when combined with WS, although this was less pronounced for runcaciguat. Neither BAY-747 or runcaciguat enhanced total GluA1 protein levels, indicating that this increased AMPAR mobilization originated from a pre-existing pool of GluA1-containing AMPARs, a mechanism which has been previously described^52^. Additionally, runcaciguat but not BAY-747 enhanced the phosphorylation of GluA1 on S845, a PKG/PKA mediated phosphorylation site that increases the mobilization of GluA1 to the cell surface^53^. This suggests that only sGC activator runcaciguat enhances GluA1-containing AMPAR mobilization to the surface via pS845-mediated translocation, while BAY-747 may induce GluA1-mobilization via different mechanisms, which is in concordance with previous research with sGC stimulator vericiguat^13^.

To further investigate the effects of BAY-747 and runcaciguat on hippocampal plasticity, C57BL/6 mice were treated with either 1.0 mg/kg BAY-747, 1.0 mg/kg runcaciguat, or vehicle p.o. 30 min before the T1 of an OLT. 24 h after the T1, the mice were sacrificed, and plasticity markers were measured in the hippocampus of these mice to reflect the hippocampal plasticity state of the mice during long-term memory performance testing. 1.0 mg/kg runcaciguat enhanced GluA1 mobilization to the surface in vivo as also shown in the ex vivo cLTP model, yet this effect was much longer lasting, i.e. for 24 h. The effects of the sGC stimulator BAY-747 were less pronounced and not as long-lasting as for runcaciguat, hence no effects of BAY-747 on GluA1 mobilization to the surface was found in vivo. The effects of runcaciguat are similar to the effects of PDE5 inhibitor vardenafil in an identical model for measuring in vivo AMPAR dynamics in a study by Argyrousi et al.^20^, which was the first study to describe a long-lasting effect of enhanced cGMP signalling on GluA1-AMPAR mobilization.

Contrary to the AMPAR results, both BAY-747 and runcaciguat were found to affect 24 h long-lasting BDNF cleavage dynamics; both 1.0 mg/kg BAY-747 treatment and 1.0 mg/kg runcaciguat treatment enhanced the ratio of mBDNF/16 kDa proBDNF, while the protein levels of the unspliced 32 kDa full BDNF remained unaltered. This suggests that the increased presence of mBDNF in comparison to the proBDNF fragment was due to a shift toward mBDNF cleavage. The full length, 32 kDa BDNF fragment can be cleaved by plasmin, furin and several matrix metalloproteases (MMPs, particularly MMP-9) into 14 kDa mBDNF (for a recent review, see^23^), which was shown to be crucial for hippocampal LTP^54^. The shift toward mBDNF cleavage induced by BAY-747 and runcaciguat at the memory enhancing dose of 1.0 mg/kg p.o. would fit with the neuroprotective actions of mBDNF and involvement in LTP and research has shown that BDNF protein expression can be regulated by the NO–sGC–cGMP pathway^44,55^.

Next to BDNF, its receptor TrkB was also investigated after BAY-747 and runcaciguat treatment. Interestingly, both 1.0 mg/kg BAY-747 and 1.0 mg/kg runcaciguat reduced the ratio of full-length TrkB/truncated TrkB in the surface protein fraction, indicative of a higher truncated TrkB presence. Additionally, 1.0 mg/kg BAY-747 but not runcaciguat enhanced mobilization of truncated TrkB to the surface. Truncated TrkB was long thought to modulate mBDNF signaling negatively^56^. However, recent research has revealed a natural abundance of truncated TrkB in neurons^57^: whereas full-length TrkB is predominantly expressed during neuronal development, truncated TrkB is the most abundant TrkB neuronal isoform in adulthood^58^. Additionally, multiple neuroprotective mechanisms have been found for this TrkB isoform. Truncated TrkB is associated with distal dendritic outgrowth, while full-length TrkB is associated with proximal dendritic outgrowth^59^. Moreover, truncated TrkB plays a role in mBDNF translocation, and glial cells are known to utilize truncated TrkB surface expression as a mechanism for internalizing abundant extracellular mBDNF for storage^60^. Furthermore, truncated TrkB expression in astrocytes was found to be important for morphological outgrowth and excitatory synapse support^61^, and mediated glycine uptake resulting in prolongation of glycine-mediated synaptic activity^62^. Therefore, it can be hypothesized that the observed increase in truncated TrkB abundance could be derived from neuroglia-mediated plasticity mechanisms.

### BAY-747 and runcaciguat enter the brain to a different extent

Although both BAY-747 and runcaciguat can enter the brain, it was found that the blood brain-barrier penetration of these compounds is different. Of note, generally, 4% of the unperfused brain volume can be attributed to blood vessels, i.e. a brain penetration range > 4% indicates good brain penetration^63^. Runcaciguat and BAY-747 enter the brain in a range of 10–13% and 38–60%, respectively. As a side note, whole-body autography values can also reflect metabolites of the compound, as long as the radioactive label is not altered by the metabolism of the compound. This may explain the descrepancies between the brain-to-plasma ratio of brain penetration and the data obtained from whole-body autography, for each compound individually. Regardless, the different brain-penetrative behavior between BAY-747 and runcaciguat has to be kept in mind for the interpretation of the results. Based on the different blood–brain barrier penetration of BAY-747 > runcaciguat, it is very difficult to compare the effect size of BAY-747 and runcaciguat on neuroprotection in vivo. Indeed, in the long-term memory study, both molecules exhibited a comparable effect on memory performance. However, in the in vivo plasticity studies, different effects for runcaciguat and BAY-747 were found. Here, it should be kept in mind that the brain concentration of the molecules is still only one aspect, i.e. amount of free fraction, enzyme affinity and enzyme stimulation/activation (IC50s) are also additional factors to consider of a molecule. Thus, it cannot be finally concluded if sGC stimulators or sGC activators as such are superior to the other.

## Conclusions

The sGC stimulator BAY-747 and sGC activator runcaciguat are both brain penetrant and enhance long-term memory acquisition processes in rats with a wide therapeutic window. Interestingly, only sGC stimulator BAY-747 was able to attenuate short-term memory deficits induced by NOS-inhibitor L-NAME. Furthermore, BAY-747 and runcaciguat both enhanced GluA1-AMPAR trafficking ex vivo in a cLTP model for memory acquisition, similar to the mechanistics of non-penetrant sGC stimulator vericiguat. In vivo, BAY-747 and runcaciguat had slightly differential mechanistic effects on long-lasting hippocampal plasticity underlying memory acquisition processes: the effects of sGC stimulator BAY-747 on the neurotrophic system were more pronounced, while runcaciguat particularly had long-lasting effects on the AMPAR system with less pronounced effects on the neurotrophic system. Altogether, the results indicate that both sGC stimulators and activators hold potential as cognitive enhancers, while the effects may not differ only on a drug mechanistic basis, but also on a plasticity mechanistic basis. This may have important implications for the choice of either sGC stimulation or sGC activation in the treatment of cognitive impairments in specific neurodegenerative diseases.

## Supplementary Information

Supplementary Information 1.

Supplementary Information 2.

Supplementary Information 3.

## Data Availability

All data analysed during the current study are available from the corresponding author on reasonable request.
