# Supplementary material for: The sGC stimulator BAY-747 and activator runcaciguat can enhance memory in vivo via differential hippocampal plasticity mechanisms
Source: Sci Rep. 2022 Mar 4;12:3589. doi: 10.1038/s41598-022-07391-1 (PMC8897390; doi:10.1038/s41598-022-07391-1)
Supplement: Supplementary file 1 — Supplementary Information 1. [file 41598_2022_7391_MOESM1_ESM.docx]

The sGC stimulator BAY-747 and activator runcaciguat can enhance memory in vivo via differential hippocampal plasticity mechanisms

Ellis Nelissen^1*^, Nina Possemis^1^, Nick P. van Goethem^1^, Melissa Schepers^2^, Danielle A. J. Jongen^1^, Lisa Dietz^3^, Wiebke Janssen^3^, Michael Gerisch^3^, Jörg Hüser^3^, Peter Sandner^3,4^, Tim Vanmierlo^2^, Jos Prickaerts^1*^

^1^ Department of Psychiatry and Neuropsychology, School for Mental Health and Neuroscience (MHeNS), Maastricht University, Universiteitssingel 50, 6229 ER, Maastricht, The Netherlands

^2^ Neuro-immune connect and repair lab, Biomedical Research Institute, Hasselt University, Hasselt 3500, Belgium.

^3^ Bayer AG, Pharmaceuticals R&D, Pharma Research Center, 42113 Wuppertal, Germany

^4^  Hannover Medical School, 30625 Hannover, Germany

* corresponding authors at: e.nelissen@maastrichtuniversity.nl and jos.prickaerts@maastrichtuniversity.nl

**Supplemental Methods**

***Chemical LTP set-up***

**
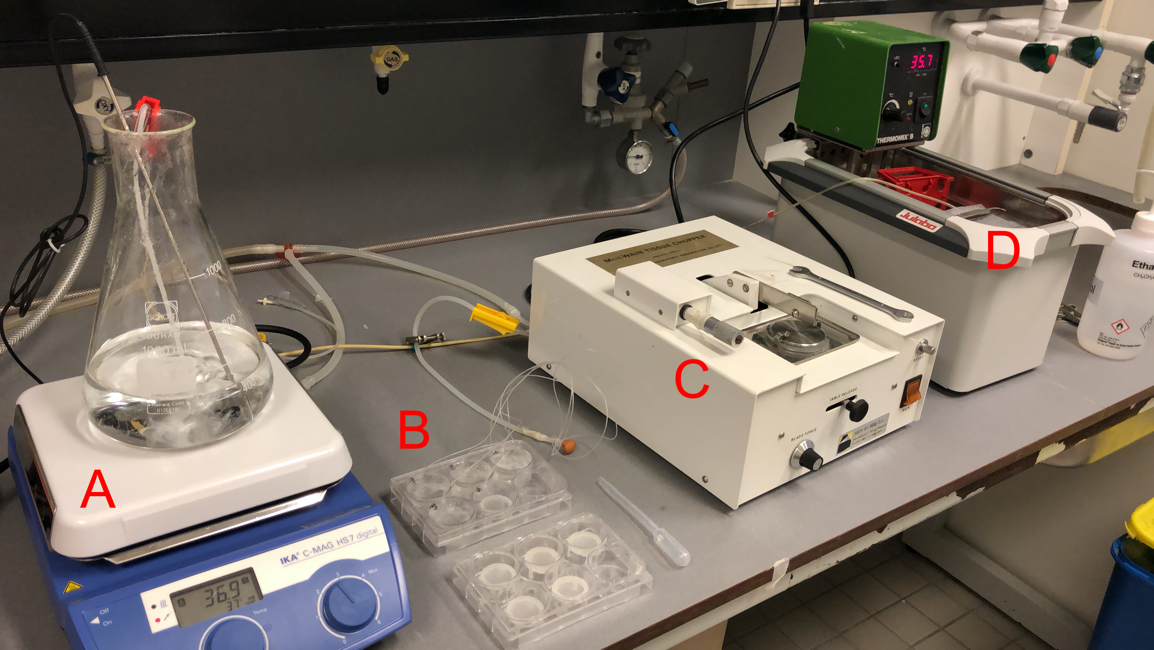
**

Supplemental Methods Figure M1. Overview of the chemical LTP set-up. A) Artificial CSF is controlled at a temperature of 37^o^C under constant oxygenation. B) Drug incubation chambers, C) McIlwain tissue chopper, and D) water bath with recovery chamber covered in parafilm to prevent evaporation. Of note, the water bath temperature is consistently kept on 37^o^C and measured by a thermometer (not included in the picture).


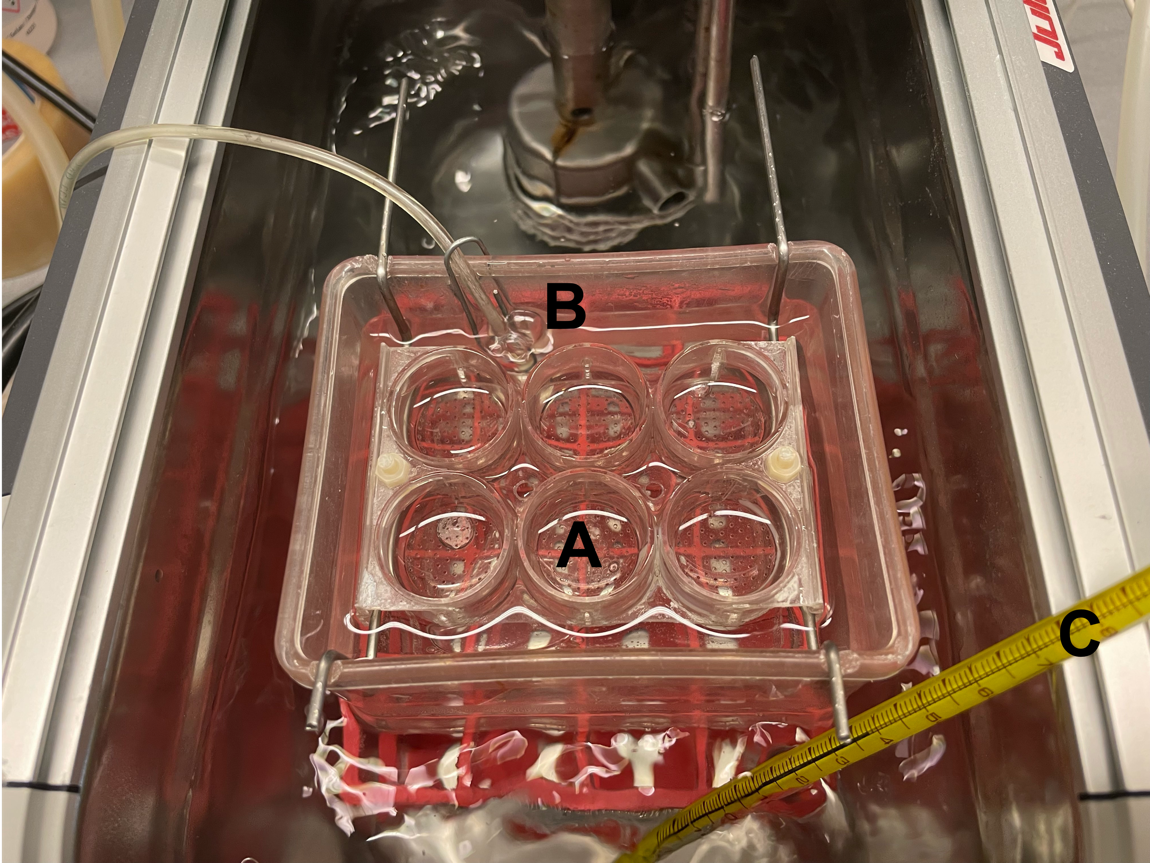


Supplemental Methods Figure M2. Close-up of the recovery chamber in the water bath. A) individual (perforated) recovery chambers filled with ACSF under B) constant oxygenation, and C) temperature controlled with an accurate thermometer at 37^o^C.


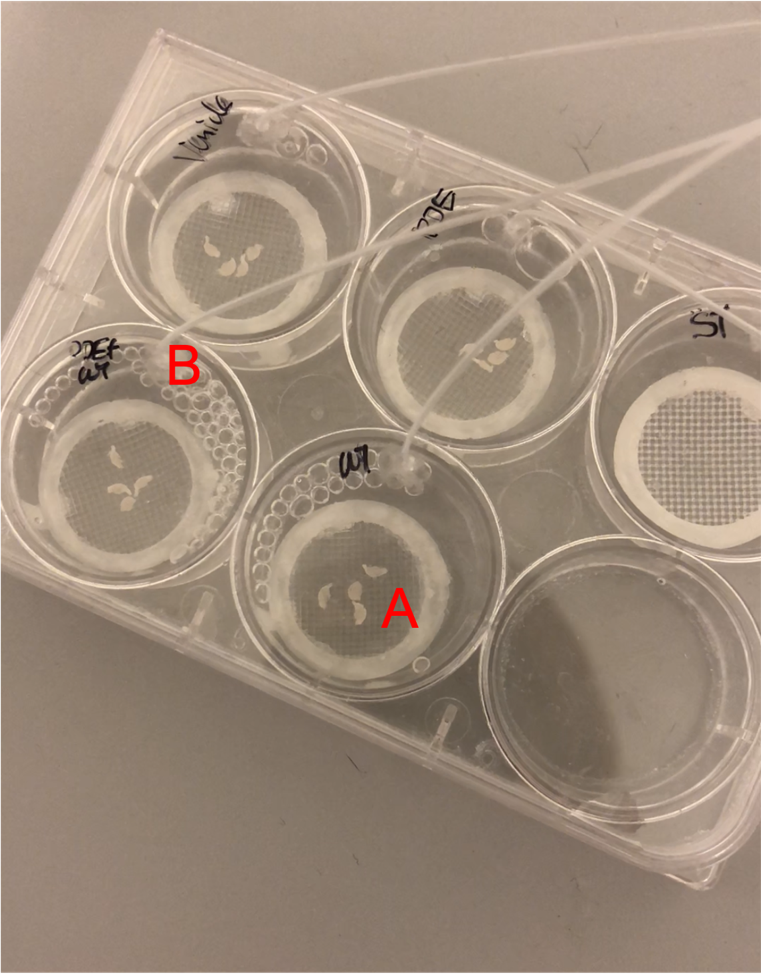


Supplemental Methods Figure M3. Close-up of the modified 6-wells plate (Costar) used for drug incubation. A) a thin grid is used in each individual well to allow for ACSF coverage of the slices, which prevents damage by reducing the movement of the slices in the ACSF. B) Modified lid of the 6-well plate with a tubing system to allow constant oxygenation of the ACSF.
