# Supplementary material for: The sGC stimulator BAY-747 and activator runcaciguat can enhance memory in vivo via differential hippocampal plasticity mechanisms
Source: Sci Rep. 2022 Mar 4;12:3589. doi: 10.1038/s41598-022-07391-1 (PMC8897390; doi:10.1038/s41598-022-07391-1)
Supplement: Supplementary file 2 — Supplementary Information 2. [file 41598_2022_7391_MOESM2_ESM.docx]

The sGC stimulator BAY-747 and activator runcaciguat can enhance memory in vivo via differential hippocampal plasticity mechanisms

Ellis Nelissen^1*^, Nina Possemis^1^, Nick P. van Goethem^1^, Melissa Schepers^2^, Danielle A. J. Jongen^1^, Lisa Dietz^3^, Wiebke Janssen^3^, Michael Gerisch^3^, Jörg Hüser^3^, Peter Sandner^3,4^, Tim Vanmierlo^2^, Jos Prickaerts^1*^

^1^ Department of Psychiatry and Neuropsychology, School for Mental Health and Neuroscience (MHeNS), Maastricht University, Universiteitssingel 50, 6229 ER, Maastricht, The Netherlands

^2^ Neuro-immune connect and repair lab, Biomedical Research Institute, Hasselt University, Hasselt 3500, Belgium.

^3^ Bayer AG, Pharmaceuticals R&D, Pharma Research Center, 42113 Wuppertal, Germany

^4^  Hannover Medical School, 30625 Hannover, Germany

* corresponding authors at: e.nelissen@maastrichtuniversity.nl and jos.prickaerts@maastrichtuniversity.nl

**Supplemental western blot figures**

1. ***Chemical LTP BAY-747***

Supplemental WB Figure A1: 6 gels (scanned together) used for the analysis of surface/total GluA1 ratios (IR 700 channel) for Figure 3 and their corresponding pS845 bands (IR 800 channel).

Supplemental WB Figure A2: 12 gels (scanned in 2 batches of 6) used for the analysis of surface/total GluA1 ratios (IR 700 channel) for Figure 3 and their corresponding pS845 bands (IR 800 channel).

Supplemental WB Figure A3: 9 gels (scanned together) used for the analysis of surface/total GluA1 ratios (IR 700 channel) for Figure 3 and their corresponding pS845 bands (IR 800 channel). Of note, after the blotting process, the methanol used for the transfer buffer was discovered to be tainted. Therefore, many membranes developed with smears or incomplete transfer processes. These membranes were not analyzed.

1. ***Chemical LTP Runcaciguat***

Supplemental WB Figure B1: 2 gels (scanned together) used for the analysis of surface/total GluA1 ratios (IR 700 channel) for Figure 3 and their corresponding pS845 bands (IR 800 channel). Of note, these membranes were scanned in parallel with another experiment (not related to the current study). Therefore, some membranes were cropped off. All membranes for runcaciguat are shown in full.

Supplemental WB Figure B2: 4 gels (scanned in batches of 2) used for the analysis of surface/total GluA1 ratios (IR 700 channel) for Figure 3 and their corresponding pS845 bands (IR 800 channel). Of note, these membranes were scanned in parallel with another experiment (not related to the current study). Therefore, some membranes were cropped off. All membranes for runcaciguat are shown in full.

Supplemental WB Figure B3: 4 gels (scanned in batches of 2) used for the analysis of surface/total GluA1 ratios (IR 700 channel) for Figure 3 and their corresponding pS845 bands (IR 800 channel). Of note, these membranes were scanned in parallel with another experiment (not related to the current study). Therefore, some membranes were cropped off. All membranes for runcaciguat are shown in full.

Supplemental WB Figure B4: 3 gels (scanned in two batches) used for the analysis of surface/total GluA1 ratios (IR 700 channel) for Figure 3 and their corresponding pS845 bands (IR 800 channel). Of note, these membranes were scanned in parallel with another experiment (not related to the current study). Therefore, some membranes were cropped off. All membranes for runcaciguat are shown in full.

Supplemental WB Figure B5: 3 gels (scanned in two batches) used for the analysis of surface/total GluA1 ratios (IR 700 channel) for Figure 3 and their corresponding pS845 bands (IR 800 channel). Of note, these membranes were scanned in parallel with another experiment (not related to the current study). Therefore, some membranes were cropped off. All membranes for runcaciguat are shown in full.

1. ***In vivo memory acquisition processes***

Supplemental WB Figure C1: 4 gels used for the analysis of surface/total GluA1 ratios (IR 700 channel) for Figure 4. Of note, due to the incomplete GAPDH on membrane 3 in the initial scan, the membrane was rescanned.

Supplemental WB Figure C2: 2 gels used for the analysis of BDNF ratios (IR 800 channel) for Figure 5, and the corresponding GAPDH (IR 700 channel).

Supplemental WB Figure C3: 4 gels used for the analysis of TrkB ratios (IR 800 channel) for Figure 5, and the corresponding GAPDH (IR 700 channel). Of note, GluA1 was also stained, but was not analyzed from these membranes due to some artifacts in the signal.
