# Supplementary material for: The sGC stimulator BAY-747 and activator runcaciguat can enhance memory in vivo via differential hippocampal plasticity mechanisms
Source: Sci Rep. 2022 Mar 4;12:3589. doi: 10.1038/s41598-022-07391-1 (PMC8897390; doi:10.1038/s41598-022-07391-1)
Supplement: Supplementary file 3 — Supplementary Information 3. [file 41598_2022_7391_MOESM3_ESM.docx]

The sGC stimulator BAY-747 and activator runcaciguat can enhance memory in vivo via differential hippocampal plasticity mechanisms

Ellis Nelissen^1*^, Nina Possemis^1^, Nick P. van Goethem^1^, Melissa Schepers^2^, Danielle A. J. Jongen^1^, Lisa Dietz^3^, Wiebke Janssen^3^, Michael Gerisch^3^, Jörg Hüser^3^, Peter Sandner^3,4^, Tim Vanmierlo^2^, Jos Prickaerts^1*^

^1^ Department of Psychiatry and Neuropsychology, School for Mental Health and Neuroscience (MHeNS), Maastricht University, Universiteitssingel 50, 6229 ER, Maastricht, The Netherlands

^2^ Neuro-immune connect and repair lab, Biomedical Research Institute, Hasselt University, Hasselt 3500, Belgium.

^3^ Bayer AG, Pharmaceuticals R&D, Pharma Research Center, 42113 Wuppertal, Germany

^4^  Hannover Medical School, 30625 Hannover, Germany

* corresponding authors at: e.nelissen@maastrichtuniversity.nl and jos.prickaerts@maastrichtuniversity.nl

**Supplemental tables:**

The exploration times during T1 and T2 for the long-term memory acquisition experiments are summarized in Table S1. For the exploration times during T1 of the BAY-747 experiment, post-hoc LSD tests revealed that the vehicle condition showed a significantly lower object exploration when compared to the 0.1, 0.3 and 1.0 mg/kg BAY-747 conditions. Furthermore, 0.01 mg/kg BAY-747 showed a significantly lower object exploration when compared to the 1.0 mg/kg BAY-747 condition. Also, 0.03 mg/kg BAY-747 showed a significantly lower object exploration when compared to the 0.1 and 1.0 mg/kg BAY-747 conditions. Moreover, the 0.1 mg/kg BAY-747 condition showed a significantly lower object exploration when compared to the 3.0 mg/kg BAY-747 and 1.0 mg/kg donepezil conditions. 0.3 mg/kg BAY-747 showed a lower exploration activity in T1 when compared to 1.0 mg/kg donepezil. Finally, 1.0 mg/kg BAY-747 showed a significantly lower object exploration when compared to 3.0 mg/kg BAY-747 and 1.0 mg/kg donepezil in T1. For the T2, the vehicle condition showed a significantly lower object exploration when compared to all of the BAY-747 conditions (i.e. 0.01, 0.03, 0.1, 0.3, 1.0 and 3.0 mg/kg BAY1165747). For the exploration times of the runcaciguat experiment no differences were found during T1 and T2. Similarly, no effects on exploration times during T1 and T2 were found in the L-NAME experiment (Table S2).

**Supplemental Table S1. The effects of BAY-747, runcaciguat and donepezil on exploration times and memory performance in the 24 h interval OLT**

| **n** | **24** | **16** | **16** | **16** | **16** | **16** | **16** | **16** |
| --- | --- | --- | --- | --- | --- | --- | --- | --- |
|  | **Vehicle** | **0.01 mg/kg**  **BAY-747** | **0.03 mg/kg**  **BAY-747** | **0.1 mg/kg**  **BAY-747** | **0.3 mg/kg**  **BAY-747** | **1.0 mg/kg**  **BAY-747** | **3.0 mg/kg**  **BAY-747** | **1.0 mg/kg**  **donepezil** |
| **e1** | 43.81 (1.63) | 49.68 (2.84) | 46.61 (2.14) | 53.92 (2.69) | 52.96 (2.32) | 58.44 (2.22) | 46.66 (2.79) | 42.84 (2.84) |
| **e2** | 36.34 (1.54) | 48.16 (3.63) | 49.75 (2.47) | 44.88 (2.29) | 47.04 (3.34) | 47.06 (3.28) | 50.70 (4.37) | 43.34 (2.11) |
| **d2** | 0.03 (0.05) | 0.06 (0.03) | 0.20 (0.04) | 0.27 (0.06) | 0.24 (0.05) | 0.22 (0.06) | 0.04 (0.06) | 0.28 (0.04) |
| **n** | **24** | **16** | **16** | **16** | **16** | **16** | **16** | **16** |
|  | **Vehicle** | **0.01 mg/kg**  **runcaciguat** | **0.03 mg/kg**  **runcaciguat** | **0.1 mg/kg**  **runcaciguat** | **0.3 mg/kg**  **runcaciguat** | **1.0 mg/kg**  **runcaciguat** | **3.0 mg/kg**  **runcaciguat** | **1.0 mg/kg**  **donepezil** |
| **e1** | 51.98 (1.75) | 58.53 (2.23) | 57.07 (2.71) | 53.89 (3.24) | 53.96 (1.85) | 54.40 (2.48) | 56.27 (2.51) | 54.95 (4.66) |
| **e2** | 48.64 (1.44) | 52.68 (2.65) | 56.54 (4.15) | 56.37 (3.65) | 56.58 (3.63) | 48.95 (2.86) | 57.72 (2.36) | 57.26 (2.77) |
| **d2** | 0.05 (0.04) | 0.03 (0.05) | 0.12 (0.05) | 0.21 (0.05) | 0.23 (0.05) | 0.25 (0.04) | 0.08 (0.06) | 0.29 (0.05) |

Total exploration times during T1 and T2, including the discrimination index, for both BAY-747 and runcaciguat long-term memory experiments. The different doses of BAY-747, runcaciguat, and donepezil were administered 30 min before T1. The delay interval between the first and second trial was 24 h. Data represent mean (SEM).

| **n** | **23** | **24** | **12** | **12** | **12** | **12** |
| --- | --- | --- | --- | --- | --- | --- |
|  | **Vehicle + Vehicle** | **Vehicle +  L-NAME** | **0.3 mg/kg BAY-747 + L-NAME** | **1.0 mg/kg BAY-747 + L-NAME** | **0.3 mg/kg runcaciguat + L-NAME** | **1.0 mg/kg runcaciguat + L-NAME** |
| **e1** | 20.32 (1.65) | 20.13 (1.63) | 24.95 (3.42) | 24.38 (3.11) | 19.61 (1.85) | 19.16 (2.26) |
| **e2** | 21.94 (1.48) | 17.63 (1.68) | 20.23 (2.64) | 21.51 (2.38) | 18.73 (1.34) | 22.06 (1.92) |
| **d2** | 0.19 (0.05) | 0.04 (0.05) | 0.30 (0.06) | 0.16 (0.04) | 0.09 (0.08) | 0.11 (0.09) |

**Supplemental Table S2. The effects of L-NAME, BAY-747, and runcaciguat on the exploration times and discrimination index d2 in a 1 h interval OLT**

Total exploration times during T1 and T2, including the discrimination index, for L-NAME, BAY-747 and runcaciguat in a short-term memory paradigm. The different doses of BAY-747 and runcaciguat were administered p.o. acutely 30 min before T1. L-NAME was administered at 30 mg/kg p.o. for 6 consecutive days. On days 5 and 6, behavioral testing was performed and L-NAME was administered 1 h before T1. The delay interval between the first and second trial was 1 h. Data represent mean (SEM).

**Supplemental Figures:**

**Supplemental Figure S1. Concentration-response curves for BAY-747 and runcaciguat in an ex vivo cLTP model for memory acquisition processes.** A) the optimum concentration for enhancing GluA1-AMPAR trafficking in combination with weak stimulation (WS) was determined to be 100 nM for BAY-747. B) For runcaciguat, the optimum concentration was determined to be 10 nM in combination with WS. Data are represented as mean + SEM. n=3-4.

**Supplemental Figure S2. The effects of BAY-747 and runcaciguat treatment on surface GluA1 and total GluA1 protein levels in an ex vivo cLTP model for memory acquisition processes.** BAY-747 affected neither the A) surface GluA1 levels nor the B) total GluA1 levels. BAY-747 was incubated at 100 nM. Similar results were found for runcaciguat on C) surface GluA1 and D) total GluA1 protein levels. Runcaciguat was incubated at 10 nM. Data are represented as mean + SEM.

**Supplemental Figure S3. The effects of BAY-747 and runcaciguat treatment on surface GluA1 and total GluA1 protein levels during memory acquisition processes in vivo.** A) neither 1.0 mg/kg BAY-747 p.o. nor 1.0 mg/kg runcaciguat p.o. treatment affected the surface GluA1-containing AMPAR protein levels, nor the B) total GluA1-containing AMPAR protein levels in the hippocampus of mice. Data are represented as mean+SEM.

**Supplemental Figure S4. The effects of BAY-747 and runcaciguat treatment on full-length 140kDa TrkB during memory acquisition processes in vivo.** Neither 1.0 mg/kg BAY-747 p.o. nor 1.0 mg/kg runcaciguat p.o. treatment affected the A) mobilization of full-length TrkB receptors to the surface or B) the total amount of full-length TrkB protein levels measured in the hippocampus of mice. Data are represented as mean+SEM.
